# Sterol biosynthesis, brain development, and disease

**DOI:** 10.1172/JCI208000

**Published:** 2026-07-15

**Authors:** Eric S. Peeples, Zeljka Korade, Karoly Mirnics

**Affiliations:** 1 Department of Pediatrics, University of Nebraska Medical Center, Omaha, Nebraska, USA.; 2 Child Health Research Institute, Omaha, Nebraska, USA.; 3 Division of Neonatology, Children’s Nebraska, Omaha, Nebraska, USA.; 4 Munroe-Meyer Institute for Genetics and Rehabilitation, University of Nebraska Medical Center, Omaha, Nebraska, USA.

## Abstract

Cholesterol biosynthesis is indispensable for CNS development and function. The developing brain relies almost entirely on intrinsic sterol synthesis to support membrane biogenesis, axonal outgrowth, synaptogenesis, and myelination. Pathogenic variants in sterol biosynthetic enzymes, including DHCR7 and DHCR24, result in complex neurodevelopmental disorders such as Smith-Lemli-Opitz syndrome and desmosterolosis. In addition to cholesterol-lowering drugs (statins), some other pharmacological agents such as antipsychotics, antidepressants, and beta blockers can also inhibit cholesterol biosynthesis due to off-target effects. This inhibition produces dual pathophysiological effects: cholesterol depletion and accumulation of its precursor, 7-dehydrocholesterol, an exceptionally oxidizable molecule that spontaneously generates toxic oxysterols. Given the intense demand for cholesterol synthesis in the developing brain, prenatal exposure to sterol biosynthesis–inhibiting medications may have far-reaching effects. In this Review, we describe convergent biochemical, genetic, and epidemiologic data that implicate developmental sterol dysregulation as a modifiable risk factor for neurodevelopmental pathology and underscore the urgent need for routine sterol pathway safety assessment in drug development and prenatal pharmacotherapy.

## Introduction

Cholesterol is a greatly misunderstood molecule. While high cholesterol levels are clearly detrimental for cardiovascular health (1), this Janus-like molecule gets an undeservedly bad reputation (2). Cholesterol is a structural component of all mammalian cells, particularly those in the brain. Although in humans the brain constitutes just 2% of total body weight, it contains up to 25% of the body’s cholesterol and cholesterol derivatives (3). Nearly all brain cholesterol is synthesized locally and exists in its unesterified form (3, 4). Sterol biosynthesis pathways generate precursors for many biological processes (5, 6). Furthermore, recent studies identified over 250 cholesterol-binding proteins, including receptors, channels, and enzymes involved in sugar regulation, glycerolipid maintenance, vesicular trafficking, protein glycosylation and degradation, and glycine transport (7–11). In the brain alone, cholesterol is involved in cell division (12, 13), membrane raft assembly (14), neuronal morphology (15), neurotransmitter release (16), myelin sheath growth and repair (17, 18), and many other critical homeostatic processes (19, 20). Due to its considerable role in brain structure and function, alterations in sterol biosynthesis throughout development can result in severe neurodevelopmental effects (21, 22). Here, we review the critical functions of cholesterol in neurodevelopment and the potential consequences of genetic factors and medications — specifically sterol biosynthesis–inhibiting medications (SBIMs) (23) — that disrupt cholesterol biosynthesis.

## Cholesterol biosynthesis

**Figure 1:**
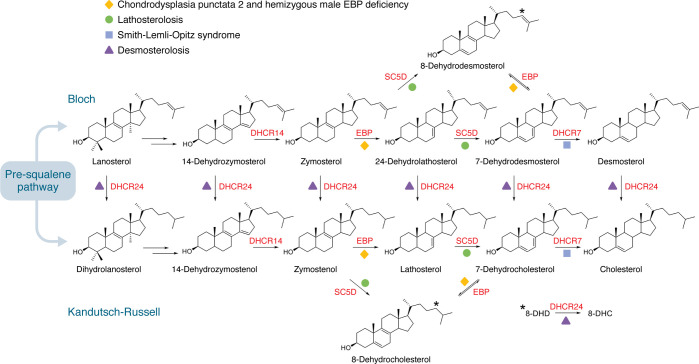
Enzymes and intermediates of the postlanosterol biosynthetic pathway. Enzymes are denoted in red font; sterol intermediates are in black. Bloch branch intermediates are in the top row; Kandutsch-Russell branch intermediates are in the bottom row. Colored shapes denote genetic inhibition associated with four inborn errors of postlanosterol biosynthesis (also see Table 1).

At the molecular level, developmental production of cholesterol involves complex, coordinated expression of enzymes across the two interconnected sterol biosynthesis pathways (Kandutsch-Russell and Bloch branches) (6, 7) (Figure 1). The condensation of acetyl-CoA units into mevalonate, catalyzed by HMG-CoA reductase, is the rate-limiting step and a major point of control. Subsequent reactions yield lanosterol, which then undergoes multiple postsqualene modifications mediated by lanosterol synthase (LSS), 24-dehydrocholesterol reductase (DHCR24), 7-dehydrocholesterol reductase (DHCR7), and other sterol isomerases and reductases. During perinatal brain development, expression of DHCR24 and DHCR7 is particularly elevated, consistent with the high demand for cholesterol in axonal growth and synaptogenesis (24). Transcriptional regulation of these enzymes is further modulated by multiple SREBPs, liver X receptors, and other lipid-sensitive transcription factors (25–29).

Sterol biosynthesis can be perturbed through multiple mechanistic pathways. Transcriptional downregulation of sterol biosynthesis genes — via suppression of SREBP or other regulatory transcription factors — can reduce pathway capacity (9, 25, 30, 31). Direct enzymatic inhibition of postsqualene reductases and isomerases can further disrupt both the Kandutsch-Russell and Bloch branches (32). Perturbations of NADPH availability can impair essential reductive steps, while mitochondrial dysfunction or oxidative stress may exert secondary inhibition across the pathway (33–35). Decreased flux through the mevalonate pathway, as well as competition for cytochrome P450 enzymes that modify sterols and oxysterols, can additionally shift metabolite pools. All of these mechanisms can destabilize sterol homeostasis and alter downstream sterol accumulation and/or oxysterol signaling.

## Cholesterol and the developing brain

Cholesterol biosynthesis in the developing CNS is a tightly orchestrated process essential for neural proliferation, differentiation, and structural maturation (3). Once the blood-brain barrier forms, isolating the CNS from circulating lipoprotein-bound cholesterol, the embryonic and postnatal brain depends almost entirely on intrinsic sterol synthesis (3, 36). In humans, sterol biosynthesis starts in the fetal brain at 19–20 weeks of gestation and peaks in the early postnatal years (37).

Most of what we know about cholesterol synthesis and function during brain development derives from studies of human disease and mouse models with deficiencies in sterol biosynthetic enzymes. Developing neurons, astrocytes, and oligodendrocytes are highly active cholesterol synthesizers both in vitro and in vivo (38–40). Cortical neurons not only produce cholesterol endogenously but also take up ApoE-complexed cholesterol produced by glial cells (41), and impairments in either biosynthesis or uptake can disrupt neuronal maturation. Production of myelin membranes requires exceptionally high cholesterol levels, and experimental suppression of cholesterol synthesis markedly impairs myelin growth, leading to hypomyelination (42). Cholesterol deficiency in oligodendrocytes causes ataxia and tremor in mice (18). In chronic myelin injury models, neurons increase their own cholesterol synthesis to support repair and remyelination, underscoring the importance of neuronal-oligodendroglial cholesterol exchange (38).

Recent advances in mass spectrometry imaging have enabled spatial mapping of cholesterol in mouse brain sections, revealing region-specific abundance across white and gray matter, developmental stages, and disease states (43, 44). Cholesterol levels are highest in the pons, cerebellar white matter, corpus callosum, and brainstem, and lowest in the olfactory bulbs and cortex. The complexity of brain sterol metabolism was further illustrated by the profiling study by Meljon et al. (45), which identified not only sterols along the cholesterol pathway but also at least 27 cholesterol- or 7-dehydrocholesterol–derived (7-DHC–derived) oxysterols in whole-brain homogenates of control and *Dhcr7*-deficient newborn mice.

Cholesterol has an essential role in monoaminergic neurotransmission. Proper development of the serotonergic (5-hydroxytryptamine [5-HT]) system depends on intact cholesterol biosynthesis. For example, in the *Dhcr7*-KO mouse model, 5-HT immunoreactivity is markedly increased in the hindbrain (46). A less severe compound *Dhcr7*-deficient transgenic mouse model (Δ3-5/T93M) shows elevated expression of the serotonin transporter and increased 5-HT uptake in isolated synaptosomes (47). Strikingly, cholesterol-dependent alterations in serotonergic signaling may also be present in *Dhcr7* heterozygotes: aged adult mice exhibit heightened head-twitch responses indicative of altered 5-HT2A receptor function (48). At the molecular level, simulations show that cholesterol binds directly to conserved sites on the dopamine transporter, stabilizing its outward-facing conformation (49). Cholesterol also modulates ligand binding and G protein coupling of the 5-HT1A receptor, an effect not rescued by 7-DHC substitution (50).

Cholesterol also plays a role in many other fundamental developmental homeostatic processes in the brain. Transcriptomic profiling of *Dhcr7*-KO astrocytes revealed an immune-activation signature, including increased glial fibrillary acidic protein expression, cellular hypertrophy, and altered calcium dynamics. These changes were driven by microglial signaling (51). Induced pluripotent stem cell–derived neurons from DHCR7-deficient patients show downregulation of WNT/β-catenin signaling and precocious neuronal specification (52). Concordantly, 7-DHC–derived oxysterols promote premature neurogenesis and depletion of neural precursor pools (53). The 7-DHC–derived oxysterol DHCEO induces aberrant crosstalk between the glucocorticoid receptor and the neurotrophin receptor TrkB, leading to hyperactivation of the MEK-ERK-C/EBP pathway in cortical precursors. Cholesterol is also indispensable for Hedgehog (Hh) signaling, a key developmental morphogen pathway (54). Cholesterol is required for Hh ligand processing, signal reception, and downstream transduction (55). Accordingly, as described below, alterations to cholesterol biosynthesis during development, whether genetic or environmental, can have devastating consequences.

## Sterol biosynthesis gene variants result in neurodevelopmental disorders

Inborn errors of cholesterol metabolism encompass a group of rare but severe genetic disorders resulting from loss-of-function pathogenic variants (mutations) that impair specific enzymatic steps in the sterol biosynthetic pathway (22). The spectrum of clinical severity correlates with residual enzymatic activity and the degree of sterol intermediate accumulation. Notable examples of inborn errors of sterol biosynthesis include Smith-Lemli-Opitz syndrome (SLOS; DHCR7 deficiency) (56), desmosterolosis (DHCR24 deficiency) (57), lathosterolosis (*SC5D* pathogenic variants) (58), squalene synthase deficiency (59), LSS deficiency (60), X-linked chondrodysplasia punctata 2 (61–63), and 17β-hydroxysteroid dehydrogenase type 7 deficiency (64–66). These diseases are characterized by distinct biochemical profiles and overlapping neurological and dysmorphological phenotypes.

Most of these disorders are rare, and only variants sparing some enzymatic activity are compatible with life. Clinically affected individuals typically present with a combination of craniofacial anomalies, limb malformations, cataracts, ichthyosis, skeletal dysplasias, growth restriction, and variable other organ defects (Table 1). Neurologic manifestations range from hypotonia and seizures to structural brain anomalies and intellectual disability. Biochemical diagnosis relies on sterol profiling by gas chromatography–mass spectrometry or liquid chromatography–tandem mass spectrometry, which identify characteristic precursor accumulation patterns. Genetic diagnosis is established by DNA sequencing. Treatment options remain limited and largely supportive; however, cholesterol supplementation might benefit some patients, and emerging strategies aim to reduce toxic sterol intermediates or enhance residual enzymatic activity (67, 68).

Desmosterol is the immediate precursor to cholesterol in the Bloch pathway, and it is used as a marker of endogenous cholesterol synthesis. It also serves as a ligand for liver X receptors. Desmosterol is converted to cholesterol by DHCR24 (Figure 1). Desmosterolosis is a rare autosomal recessive disorder caused by biallelic pathogenic variants in *DHCR24* (69–71). Pathogenic variants lead to markedly reduced enzyme activity and consequent accumulation of desmosterol in plasma and tissues. Clinically, affected individuals present with a constellation of multiple congenital anomalies, including corpus callosum agenesis or hypoplasia, white matter loss, microcephaly or macrocephaly, arthrogryposis, spasticity, cleft palate, facial dysmorphism, ocular abnormalities, and seizures (72). Intellectual disability, delayed motor development, and failure to thrive are reported across all cases. Due to its rarity, phenotypic overlap with inborn errors of postlanosterol biosynthesis, and limited availability of testing, many cases might never be diagnosed.

SLOS is the most common and best characterized inborn error of sterol biosynthesis, caused by homozygous loss-of-function pathogenic variants in *DHCR7*, which encodes the enzyme responsible for reducing 7-DHC to cholesterol in the final biosynthetic step (56). SLOS manifests with microcephaly, intellectual disability, dysmorphic features, and behavioral disturbances. SLOS can be considered a sonic hedgehog (SHH) pathway disease because the SHH protein needs cholesterol to become active, and without sufficient cholesterol, the SHH signaling pathway is disrupted, leading to many of the developmental abnormalities associated with the disorder (73). In mouse models of SLOS and lathosterolosis, reduced cellular cholesterol correlates with diminished Hh responsiveness (55). Sever et al. (74) identified B-ring 7-DHC–derived oxysterols that inhibit Hh signaling by blocking the Hh receptor Smoothened, providing a mechanistic link between sterol biosynthesis defects and impaired morphogen signaling.

Unique to SLOS is the accumulation of 7-DHC oxysterols that have biological activity and likely contribute to the phenotype seen in this disorder (75, 76). Notably, many SLOS patients exhibit a behavioral phenotype consistent with autism spectrum disorder (ASD) (77). Furthermore, in humans with SLOS, cerebrospinal fluid levels of serotonin metabolite 5-HIAA and dopamine metabolite homovanillic acid are reduced relative to age-matched controls (78), suggesting a sterol-related defect in synaptic vesicle formation and neurotransmitter release.

Beyond SLOS and other inborn errors of sterol biosynthesis, additional neurodevelopmental and autism-related disorders have also been linked to disruptions in cholesterol metabolism. In a small study of children with ASD, 19% (19/100) had serum cholesterol levels below the fifth percentile for age (79). A landmark study from the Kohane lab in 2020 found that 6.5% of individuals with ASD had abnormal lipid levels — nearly twice the number of those without autism (80). In addition, when the fathers in the families studied had lipid abnormalities, the risk of having a child with autism was 13% higher. This comprehensive study provided strong evidence for a dyslipidemia-associated ASD subtype (80). Individuals with Rett syndrome (MECP2 variants) also exhibit elevated circulating cholesterol (81, 82), whereas patients with Fragile X syndrome show reduced total cholesterol and HDL levels (83). Taken together, these studies highlight the potential that dysregulation of cholesterol homeostasis is a shared underlying mechanism in neurodevelopmental disorders.

## Sterol biosynthesis–inhibiting side effects of currently approved medications

In addition to genetic disruption of cholesterol biosynthesis, many commonly prescribed medications interfere with the pathway. These SBIMs can be broadly divided into two classes: those designed to mitigate hypercholesterolemia (e.g., statins inhibiting HMG-CoA reductase, the first enzyme in the biosynthetic pathway) (84) and those intended to have primarily nonsterol (e.g., neuropsychiatric, cardiac, metabolic) targets but that also inhibit sterol biosynthesis as a side effect. This second group includes medications such as aripiprazole, trazodone, amiodarone, cariprazine, buspirone, haloperidol, nebivolol, propranolol, and other compounds (39, 85–93). With distinct indications, mechanisms of actions, and organ system targets, these SBIMs each produce somewhat different biochemical disruptions of the sterol biosynthesis pathway.

Polypharmacy is becoming increasingly common in clinical practice. A recent review found that two or more medications were prescribed to pregnant women in up to 62% of cases (94). A critical but underrecognized issue is the potential summative effect of multiple SBIMs prescribed simultaneously (95). Even mild partial inhibition at different enzymatic steps can combine to result in depletion of cholesterol or buildup of toxic intermediates (96, 97). Indeed, in vitro and rodent studies have revealed that sterol biosynthesis–inhibiting polypharmacy can have additive or synergistic effects. For example, when aripiprazole and trazodone were coadministered in vitro to developing mouse neurons or glial cells, the combination resulted in an additive, dose-dependent increase in 7-DHC, as well as decreases in desmosterol and cholesterol (96).

Because polypharmacy is common in psychiatry, neurology, and pain management, we hypothesize that cumulative SBIM burden may exceed the threshold for physiologic compensation in the developing fetus. Moreover, sterol biosynthesis–inhibiting medications that target different organ systems (e.g., cardiovascular vs. brain) are often prescribed by physicians of different specialties (e.g., cardiologists and psychiatrists), without knowing that the combination of medications might increase the magnitude of sterol inhibition.

## Human biomaterial and epidemiological studies

The best-studied medications with sterol biosynthesis–inhibiting side effects are aripiprazole, cariprazine, and trazodone (90, 91, 95, 98–101). These medications inhibit DHCR7 enzyme activity (91), resulting in reduced levels of cholesterol and increased levels of cholesterol precursors (e.g., 7-DHC). Notably, while cholesterol is a very stable molecule, with a half-life of up to a decade in the human brain, 7-DHC is the most oxidizable lipid known in the human body (102, 103): it readily undergoes chain-propagating autoxidation, generating a complex mixture of 7-DHC–derived oxysterols. Many of these oxysterols are cytotoxic, disrupt membrane architecture, impair neuronal differentiation, and alter signaling pathways (52, 74).

The initial finding that commonly used medications can have sterol biosynthesis–inhibiting side effects was reported in 2013 by Hall et al. (99). They examined the medical records of patients with elevated blood 7-DHC to determine the origin of this biochemical disturbance and found that consumption of aripiprazole and trazodone, in the absence of *DHCR7* pathogenic variants, caused elevations of blood 7-DHC of 22 individuals, leading to misdiagnosis of SLOS in these patients (99). Further studies confirmed this elevation of 7-DHC levels in the blood of psychiatric patients taking aripiprazole, trazodone, cariprazine, haloperidol, and buspirone, as well as in the postmortem brain tissue of those taking trazodone (98, 101).

Sterol biosynthesis inhibition is also detectable in the blood of pregnant women taking prescription SBIMs. In a study of 1,312 deidentified serum samples from pregnant women, 302 showed elevated 7-DHC levels over the 95% CI of all studied samples (95). Of these, 43 had detectable levels of one or more of 14 prescription medications with a DHCR7-inhibiting side effect. Further, 7-DHC levels increased in proportion to the number of SBIM medications detected, indicating a potentially additive or synergistic effect.

Finally, a landmark study by Boland and Tatonetti in 2016 reviewed fetal outcomes subsequent to prenatal exposure to DHCR7 inhibitors (104). The study showed that first-trimester exposure to DHCR7 modulators had teratogenic effects (104). DHCR7-inhibiting chemical agents were associated with a 10.9% frequency of fetal dysmorphologies, while medicines that increase DHCR7 expression resulted in a 5.5% rate of fetal deformities. This was higher than the CDC-reported background rate of 3.0% (105) and the 0.0% rate with levothyroxine (a drug known to be safe during pregnancy). Furthermore, exposure to DHCR7 inhibitors was associated with a 6-fold increased odds of adverse fetal outcomes, including fetal malformations and congenital anomalies. Most recently, similar embryotoxicity has been reported in Xenopus, with lethality predominating in the statin group (simvastatin, pitavastatin, lovastatin, cerivastatin, and fluvastatin) and developmental delay and microcephaly primarily observed in the psychotropic drug–treated group (cariprazine, sertraline, and trazodone) (106). Based on these outcomes, Boland and Tatonetti recommended that DHCR7 activity should be considered during drug development and prenatal toxicity assessment (104).

## Sterol biosynthesis inhibition in fetal fentanyl syndrome

Many other chemicals also have the potential to inhibit sterol biosynthesis (92, 107). A 2023 study described children born with hypospadias, microcephaly, syndactyly, clubfoot, and other dysmorphologies that closely resembled the phenotype of SLOS (108). Blood of these children also showed a 7-DHC elevation, a biochemical hallmark of SLOS. However, genetic sequencing of the patients showed no pathological variants in the *DHCR7* gene, and the elevated 7-DHC levels normalized within a few weeks of birth. Subsequent review of maternal medical records revealed fentanyl misuse during pregnancy in all of the mothers. This led to two closely related hypotheses: first, that fentanyl is the cause of this new syndrome (subsequently termed fetal fentanyl syndrome [FFS]) and, second, that the likely mechanism was related to developmental sterol biosynthesis inhibition. Subsequent biochemical studies in cell lines, developing neurons and glia, and human dermal fibroblasts revealed that fentanyl was indeed a multienzyme inhibitor of the postlanosterol biosynthetic pathway (109). However, our understanding of this new disorder is still extremely limited; virtually no data exist on the effects of timing, dose, maternal genotype, comorbidities, and many other factors that would explain which children will develop FFS as a result of intrauterine exposure to nonprescription fentanyl.

## SBIM prescription during pregnancy and risk of ASD

Our recent study revealed that the children of mothers who are prescribed SBIMs during pregnancy have a higher frequency of being diagnosed with ASD in later life (23). The study queried the Epic Cosmos database of linked child and maternal health records for births between 2014 and 2023, with follow-up to December 2025. The study included 6,135,213 maternal-child dyads, nearly one-third of all births in the United States over the 10-year period. We assessed the incidence of ASD associated with maternal prescription of 14 medications with either primary sterol biosynthesis–inhibiting effects or known sterol biosynthesis–inhibiting side effects: aripiprazole, atorvastatin, bupropion, buspirone, fluoxetine, haloperidol, metoprolol, nebivolol, pravastatin, propranolol, rosuvastatin, sertraline, simvastatin, and/or trazodone. Cox proportional hazard modeling revealed that exposure to at least one SBIM during pregnancy was associated with a 1.47 times (95% CI 1.45–1.49) increased risk of an ASD diagnosis after adjusting for potential confounders. This increased to 2.33 (95% CI 2.09–2.61) higher risk when four or more SBIMs were coprescribed. Notably, of the 234,971 (3.8%) children with an ASD diagnosis, 35,152 (15.0%) of their mothers were prescribed at least one SBIM during pregnancy (23). Overall, this study suggests prenatal exposure to medications with sterol biosynthesis–inhibiting effects increases ASD risk, and this risk may be even greater if more than one of these medications is prescribed at the same time.

## Interaction between sterol inhibition and genetic vulnerability

Carriers of single pathogenic *DHCR7* variants (e.g., parents of SLOS patients) are asymptomatic and considered healthy. However, they have mildly elevated 7-DHC in peripheral tissue (91, 100), suggesting a latent vulnerability to SBIMs for the 1%–3% of the human population who carry the *DHCR7^+/–^* genotype (110). When human dermal fibroblasts of these individuals are exposed to DHCR7-inhibiting medications, they react with a much sharper rise in 7-DHC levels than cells from patients with two typical copies of the gene (100). Furthermore, studies in which pregnant mouse dams were exposed to aripiprazole, cariprazine, or trazodone suggest that the sharpest rise of 7-DHC occurs in the brain of *Dhcr7^+/–^* transgenic mouse embryos originating from *Dhcr7^+/–^* transgenic dams (111). These data suggest that developmental vulnerability to medications with sterol biosynthesis–inhibiting side effects may be greatly affected by both maternal and offspring genotype.

In summary, when both the mother and fetus are carriers of pathogenic variants of sterol biosynthesis genes, sterol synthesis inhibition resulting from utilization of multiple SBIMs during pregnancy might exceed the compensatory capacity of the developing brain, with deleterious consequences on the fetus.

## Conclusions and future directions

The work described here firmly establishes the critical requirement for cholesterol biosynthesis during brain development and indicates that exposure to SBIMs during pregnancy may have neurodevelopmental effects. The concern that medication used during pregnancy could predispose to adverse outcomes for the baby is not new (112), and medication package inserts developed by pharmaceutical companies clearly state the risks of medications if taken during pregnancy. Unfortunately, although the package inserts are easily accessible for physicians and patients, the relevant data are often buried in the large amount of information in them. Notably, in 2015, the FDA retired the A, B, C, D, and X risk pregnancy categories, which had been used since 1979 (113). The categories were replaced with narrative sections and subsections to allow for more nuance and data transparency. However, these narrative sections may cause more confusion, especially for patients with low health literacy and for physicians attempting to balance the time burden of their clinical duties with interpreting the often complex and sometimes conflicting study results listed in the inserts.

Importantly, the data from our recent study (23) suggest only one of likely several important associations with ASD and do not conflict with the extensively documented, strong genetic component of ASD (114–116). Not all pregnancies prescribed SBIMs resulted in a child with ASD, and it is not yet clear which factors may exacerbate the sterol-inhibiting effects (or side effects) of these medications. The variables that could interact with SBIM use are nearly endless, including the sterol biosynthesis gene variants of the mother and developing baby, comorbidities, polypharmacy, environmental influences, socioeconomical circumstances, and nutritional factors. Furthermore, the dose, duration, and timing of SBIM administration are likely to also be critical determinants of ASD risk.

Notably, one potential risk factor is widely overlooked. There is extremely limited data on the effects of paternal SBIM use in the context of reproductive health. Cholesterol is essential for male fertility as a precursor to testosterone and a structural component of sperm membranes (117). Sperm divide extremely fast, and animal model studies show a negative effect of aripiprazole on male fertility, sperm motility, and sperm quality (118, 119). Similarly, atorvastatin treatment has been shown to alter semen parameters in healthy males, including the total number of spermatozoa, vitality, total motility, and kinetics (120). The effect of paternal SBIM use on fetal development is thus far untested. However, given that paternal alcohol consumption before conception can disrupt offspring’s brain, skull, and face development (121), investigating the impact of environmental factors including paternal SBIM use on neurodevelopmental outcomes should become a priority (122).

It is also important to acknowledge that in addition to its neurodevelopmental roles, cholesterol is also critical for adult brain function. As in the developing brain, cholesterol is critical in the adult brain for structural integrity and to enable efficient synaptic transmission. Dysregulated cholesterol homeostasis may contribute to the development of neurodegenerative diseases, including Alzheimer’s disease (123, 124). How interventions including SBIMs may interact with this biology is another area of intense interest in the field (125).

**Figure 2:**
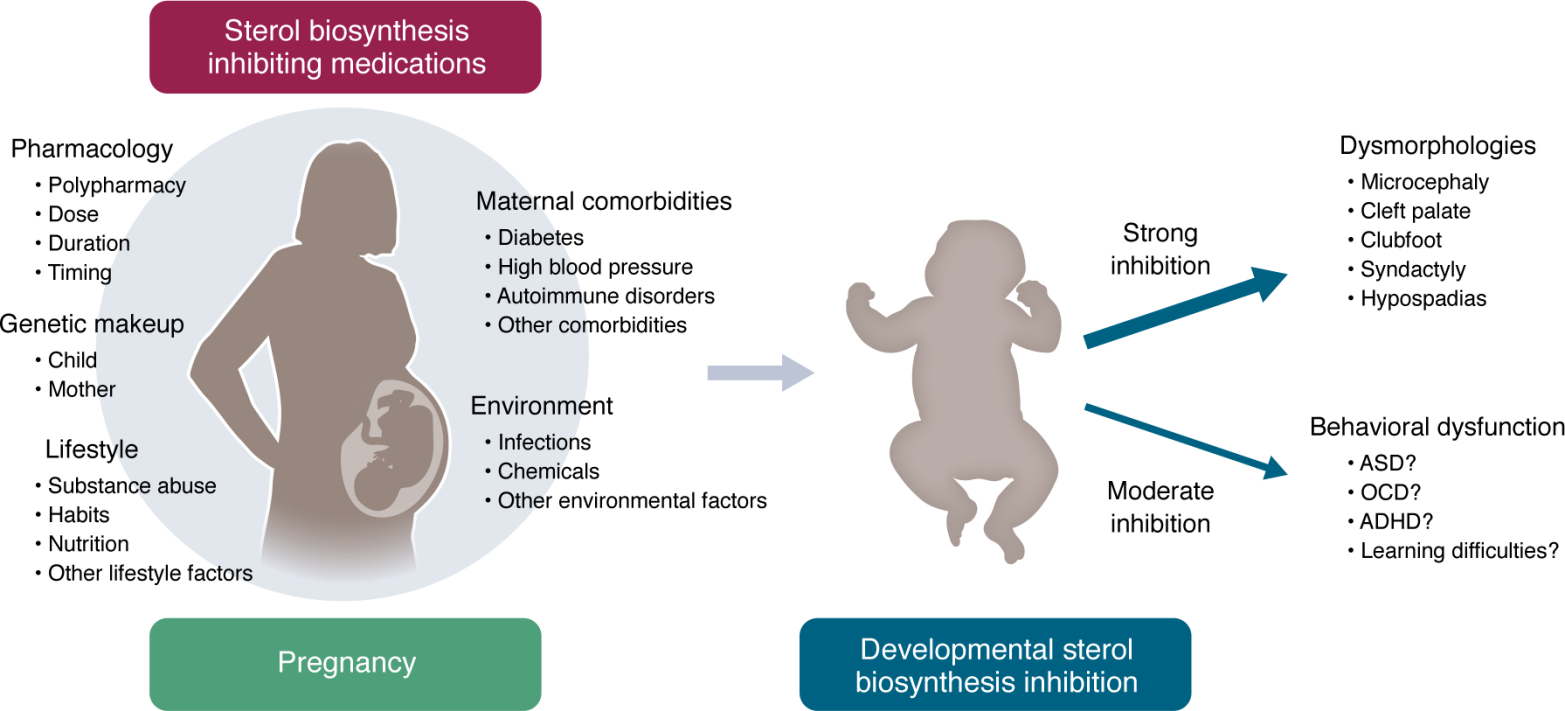
Proposed neurodevelopmental pathophysiology and consequences of developmental sterol inhibition. SBIMs utilized during pregnancy can interact with the normal developmental trajectory of the fetal brain and body. The magnitude of the sterol inhibition depends on many factors highlighted in the circle. We propose that the strongest sterol inhibition would result in SLOS-like dysmorphologies, while a more moderate inhibition would result in functional consequences with a delayed manifestation of functional connectivity changes that could persist through a lifetime. ADHD, attention-deficit/hyperactivity disorder; ASD, autism spectrum disorder; OCD, obsessive-compulsive disorder.

Finally, the combined findings above raise a critical question: Are we only seeing the proverbial tip of the iceberg? Can prenatal SBIM exposure lead to other developmental challenges, such as dysmorphologies, obsessive-compulsive disorder, learning difficulties, or anxiety later in childhood or as an adult? Does fetal sterol biosynthesis inhibition permanently rewire the developing brain, with functional/behavioral outcomes that may only reveal themselves years after the initial insult? We propose that the strongest inhibition of sterol biosynthesis might result in earlier and more clinically apparent changes (e.g., dysmorphologies), while more moderate inhibition may have primarily microanatomical and behavioral consequences (Figure 2). This complex framework deserves further investigation.

In summary, developmental cholesterol biosynthesis is fundamental for CNS maturation. The spatiotemporal regulation of sterol synthesis, enzyme expression, and intercellular lipid transport ensures adequate cholesterol supply for membrane biogenesis and signaling throughout neurodevelopment. Prenatal disruption of this finely tuned system — whether by medications or genetic, metabolic, or environmental factors — can have lasting consequences for brain structure and function. Prescription of SBIMs to pregnant women increased considerably over the period of our study, from 4.6% in 2014 to 16.8% in 2023 (23). Given the potential risks of the sterol biosynthesis–inhibiting effects, the increased utilization of SBIMs, likely reflecting increased provider comfort with prescribing these medications during pregnancy, is a cause for concern (126, 127).

## Conflict of interest

The authors have declared that no conflict of interest exists.

## Funding support

This work is the result of NIH funding, in whole or in part, and is subject to the NIH Public Access Policy. Through acceptance of this federal funding, the NIH has been given a right to make the work publicly available in PubMed Central.

- NIH grant R01MH110636 (KM)
- NIH grant R56 HD111119 (KM and ZK)
- Munroe-Meyer Institute Hattie B. Munroe Professorship (KM)
- Child Heath Research Institute funds (ESP and ZK)
- Dorothy B. Davis Foundation
- NE State Tobacco Settlement Fund allocated to UNMC

## Figures and Tables

**Table 1:**
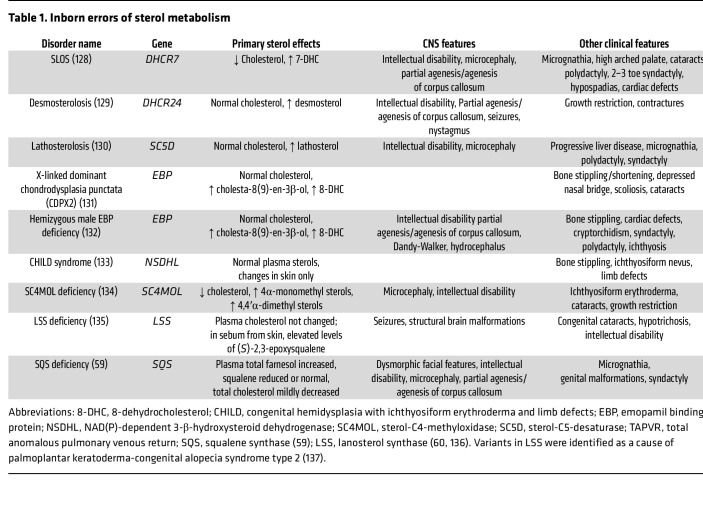
Inborn errors of sterol metabolism

## Version 1 07/15/2026

Electronic publication
